# Label-Free SERS Sensors for Real-Time Monitoring of Tyrosine Phosphorylation

**DOI:** 10.1021/acs.analchem.4c02860

**Published:** 2024-10-29

**Authors:** Ailsa Geddis, Lorena Mendive-Tapia, Audreylia Sujantho, Erica Liu, Sarah McAughtrie, Richard Goodwin, Marc Vendrell, Colin J. Campbell

**Affiliations:** † EaStCHEM School of Chemistry, The University of Edinburgh, Edinburgh EH9 3FJ, U.K.; ‡ Centre for Inflammation Research, The University of Edinburgh, Edinburgh EH16 4UU, U.K.; § IRR Chemistry Hub, Institute for Regeneration and Repair, The University of Edinburgh, Edinburgh EH16 4UU, U.K.; ∥ Clinical Pharmacology and Safety Sciences, Biopharmaceuticals R&D, AstraZeneca, Cambridge CB4 0WG, U.K.

## Abstract

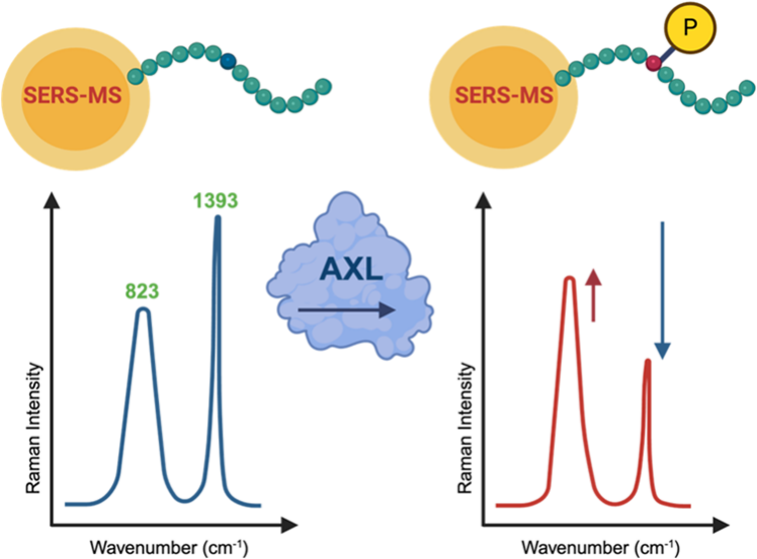

Dysregulation of receptor tyrosine kinases (RTKs) has been shown to correlate with cancer cell proliferation and drug resistance. Thus, monitoring the activity of RTKs at a chemical level could provide new biomedical insights and methods to assess the drug efficacy. However, direct monitoring of kinase activity is challenging and most commonly relies on *in vitro* techniques such as Western blotting and ELISAs. Herein, we report the development of a gold nanoparticle-based surface-enhanced Raman scattering (SERS) sensor, which allows the real-time monitoring of tyrosine phosphorylation of a reporter peptide (Axltide) by the Axl enzyme. We demonstrate that our sensor shows strong signal localization, and we are able to detect tyrosine phosphorylation of the reporter peptide through chemical phosphorylation and enzymatically with similar peak changes to those observed in the spontaneous Raman spectra. Through monitoring the SERS spectrum, we can observe changes in phosphorylation in real time.

Tyrosine phosphorylation is a common posttranslational modification that plays a critical role in cellular signaling pathways and influences various cellular processes such as cell growth, differentiation, hormone response, and immune defense.^1,2^ However, aberrant tyrosine phosphorylation has been linked with numerous diseases and become an important biomarker^3^ for various cancers including NSCLCs,^4^ breast cancer,^5^ and colorectal cancer.^6−8^ In particular, dysregulation of receptor tyrosine kinase (RTK) signaling by gene mutations can lead to developmental disorders as well as oncogenesis.^9,10^ While the mechanism behind the overexpression of some RTKs is well understood, there is a need to uncover mechanistic detail at a molecular level within pathways that are targets for cancer therapy. With such a significant impact on cancer progression, the ability to monitor Tyr phosphorylation at a molecular scale presents an important research target for advancing cancer therapy and other disease treatments, where current technologies are lacking.

Currently, the most common methods to detect tyrosine phosphorylation rely on antiphosphotyrosine monoclonal antibodies (mABs) and include Western blotting^11^ ELISA and flow cytometry assays.^12−14^ Proteomic analysis using mass spectrometry is another common approach for the detection of P.Tyr residues.^15−19^ While these methods are widely used and have proven invaluable in understanding phosphorylation events, they are not without their limitations; they often demand extensive and time-consuming preprocessing and lack the temporal and spatial resolution required for capturing dynamic phosphorylation changes.^17^ Such limitations highlight the need for new sensors able to monitor Tyr phosphorylation in real time.

Raman spectroscopy has proven to be a useful modality for providing specific information about biomolecular structure and bonding.^20^ To enhance the signal, variants such as surface-enhanced Raman spectroscopy (SERS) use plasmon resonance in various nanostructures including gold and silver nanoparticles to enhance photon scattering by up to 10^7^ and have been reported to detect single molecules.^21,22^ In particular, SERS has been successfully used to study Tyr phosphorylation by various groups. Cottat et al.^23^ examined spleen tyrosine kinase conformation by using SERS with lithography, and Guo et al.^24^ developed SERS sensors for bioimaging of tyrosine phosphorylation with artificial antibodies. Ren et al.^25^ developed SERS sensors that make use of a Raman reporter molecule and a selective peptide for kinase binding on DNA cross-linked gold nanoparticles, and Liu et al.^26^ used an AgNP-based SERS assay to detect phosphorylation activity of protein kinase A in cell extracts. While these approaches demonstrate the promising capabilities of SERS, they all require sample preprocessing often involving complex preparation techniques and either lack temporal resolution or quantitative readouts, or require fairly complex fabrication techniques. Thus, existing technologies have fallen short in managing to directly detect and quantify tyrosine phosphorylation *in situ* and in real time.

In this work, we investigate a new approach for the real-time monitoring of tyrosine phosphorylation based on SERS microsensors. Building on previous work that demonstrated that SERS microsensors (SERS-μS), based on a simple construct of a polymer microparticle decorated with gold nanoparticles, can be used to monitor pH in an *in vitro* disease model,^27,28^ we modified the functionalization to incorporate a peptide, Axltide—a substrate of various receptor tyrosine kinases (RTKs), as our reporter molecule. We found that SERS can be used to ratiometrically monitor the phosphorylation state of tyrosine in the peptide and, thus, the activity of the kinase. This approach provides an alternative method for real-time measurement and monitoring of phosphorylation.

## Experimental Section

Supporting Information regarding reagents and general methods used, peptide synthesis and characterization, and optimization assays is available online.

### Peptide Synthesis

Peptides CY, CpY, CKKSRGDYMTMQIG, and CKKSRGDpYMTMQIG were synthesized under automated microwave conditions and SPPS as detailed in the Supporting Information.

### Synthesis of SERS-Axltides (Scheme 1) [SERS-μS]

**Scheme 1:**
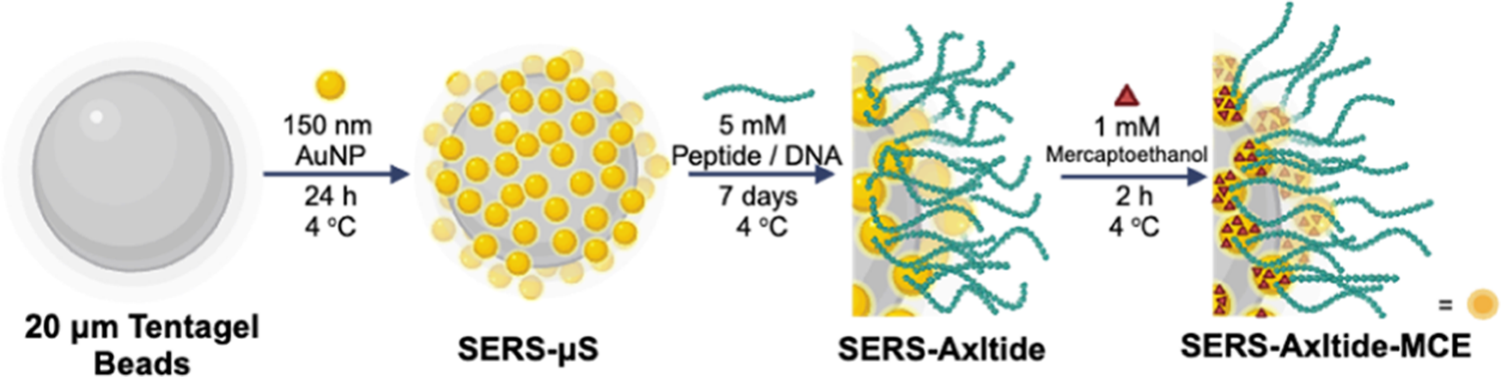
Synthesis of SERS-Axltide-MCE

150 nm gold nanoparticle (AuNP) solution in citrate buffer (3 mL) was added to 20 μm Tentagel (1 mg), sonicated for 10 min, and incubated at 4 °C for 24 h. [SERS-Axltide]. 1 mL of the resulting suspension was centrifuged (3 min at 3.0 rcf) to obtain a pellet. Buffer was removed and washed with water (3 × 1 mL). Axltide solution (5 mM, 400 μL) was added, vortexed for 1 min, sonicated for 3 min, and left to incubate at 4 °C for 1 week. [SERS-Axltide-MCE]. The bound substrate was then centrifuged to a pellet and washed three times with water. In cases where mercaptoethanol (MCE) was used as a postfunctionalization treatment, MCE solution (1 mM in water) was added to the centrifuged pellet and left to bind for 1–2 h at 4 °C and then washed with water as above. SERS-Axltide-MCE was stable for at least 2 months at 4 °C in water (Scheme 1). The background SERS spectra of both Tentagel particles and MCE are shown in Figure S3.

### Spontaneous Raman Analysis of Peptides

Raman spectra were measured using the Renishaw inVia confocal Raman microscope with a cooled CCD camera ANDOR Newton Model DU970P-FI-793 and a 785 nm Renishaw HPNIR laser with a 50× objective lens. Powder was added to a CaF_2_ slide and laser power and exposure times varied to obtain optimum signal. Cosmic ray removal was carried out on a Renishaw WiRE 5.6 software. Subsequent analysis was carried out on MATLAB_R2023a where spectra were baselined (using an ALS (Asymmetric Least Squares) GUI,^29^ where λ = 50,000, *p* = 0.0001, and iterations = 10), normalized, smoothed (Savitsky-Golay, polynomial order = 3 and framelength = 11).

### SERS Analysis of SERS-Axltide

20 μL of SERS-Axltide suspension was added to a CaF_2_ slide and covered with a glass coverslip. Circular maps of ∼50 to 150 spectra per map were taken of individual SERS-Axltide, cosmic ray removal was carried out on WiRE 5.6, and imported into MATLAB. Saturated spectra were removed, then baseline-subtracted (using an ALS (asymmetric least squares) GUI, where λ = 50,000, *p* = 0.0001, and iterations = 10). Then, background or very low-intensity spectra were removed and spectra were normalized. A mean spectrum was calculated per SERS-Axltide and smoothed (Savitsky-Golay, polynomial order = 3 and framelength = 11).

### Axl Assay General

Axl enzyme was obtained from Merck Millipore and all other reagents from Sigma-Aldrich, Merck, and Thermo Scientific. Protocol adapted from the Millipore certificate of Analysis for Axl, active (Item #14-512). Solutions were made up: 5× reaction buffer: 40 mM MOPS/NaOH pH 7.0, 1 mM EDTA; peptide solution (2.5 mM Cys-Axltide stock) or SERS-Axltide suspension (1.4 mg/mL SERS-Axltide suspension in DI water); Axl, active: dilute aliquot with 20 mM MOPS/NaOH pH 7.0, 1 mM EDTA, 0.01% Brij-35, 5% Glycerol, 0.1% 2-mercaptoethanol, 1 mg/mL BSA; and 2.5× magnesium acetate/ATP solution: solution was made up to 25 mM magnesium acetate and 0.25 mM ATP. In Eppendorf, 5 μL of reaction buffer, 5 μL of dH_2_O, 10 μL of ATP mixture, and 2.5 μL of SERS-Axltide suspension or peptide solution were added. The solutions were incubated at 30 °C for various amounts of time depending on the experiment, quenched with 5 μL of 3% phosphoric acid, and processed as above. Assays were run in triplicate.

### AXL Assay Analysis

HPLC–MS analysis was performed on an Agilent 1260/1200 HPLC instrument connected to an Agilent G6110A MS instrument with a Phenomenex column (C18, 5 μm, 4.6 × 150 mm^2^). Assay protocol as above using double quantities and injection of 20 μL into the HPLC-MS using an 8 min method with a 500–1500 *m*/*z* range from 5 to 95% ACN in H_2_O with 1% FA.

Raman analysis was performed as described above for SERS-Peptide. In preliminary assays, 3 maps were taken per replicate, and in final assays, 5 maps were taken per replicate with 50–120 spectra per map with each replicate taking approximately 5 min. A mean of this final set of spectra was taken and plotted using MATLAB. In final assay, the maximum intensity of peaks at 823 and 1393 cm^–1^ was found (±5 cm^–1^) using the “findpeaks” function on MATLAB, relative ratio was calculated, and the data was plotted on GraphPad prism 10 and analyzed using an ANOVA with Dunnett’s multiple comparisons test.

## Results and Discussion

### Synthesis and Evaluation of Axltide as a Versatile RTK Substrate for Gold Nanoparticle Conjugation

**Figure 1:**
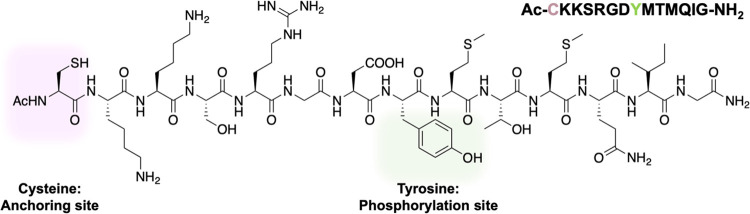
Chemical design of the Cys-Axltide peptide highlighting key residues: cysteine (pink) to achieve assembly to gold nanoparticles and tyrosine (green), where phosphorylation occurs.

Our first aim was to make a reporter molecule whose SERS spectrum could report Tyr phosphorylation. Hence, our design was based on Axltide peptide (KKSRGDYMTMQIG)—a substrate for Axl, which is a member of the TAM family of RTKs with diverse roles in immune response regulation, cell adhesion, and apoptotic signaling. In particular, Axl has been implicated in cancer progression, metastasis, and resistance to therapy in various malignancies.^30,31^ The overexpression of Axl is often associated with poor prognosis in cancer, making it an attractive therapeutic target. The peptide reporter also included an N-terminal cysteine residue as the anchoring site to the gold nanoparticles (Figure 1).

Axltide has been used as a model peptide for kinase assays in various studies with cell-surface enzymes such as DDR2,^32^ MerTK,^33^ and Axl. The wide range of kinases able to phosphorylate Axltide and the presence of only one residue for phosphorylation indicates a broad utility for this peptide as a reporter molecule.^34^ We incorporated cysteine to the N-terminus of the Axltide peptide to allow attachment to the gold surface of the SERS-μS. We believe this specific anchoring point is important to allow optimal orientation for kinase binding. Although amine-gold bonds are possible, we believe that the thiol modification improves binding strength and consistency. Details of the synthesis and characterization can be found in the Materials and Methods section of the Supporting Information.

### Raman Spectroscopy Identifies the Main Peaks Associated with Tyrosine Residue

**Figure 2:**
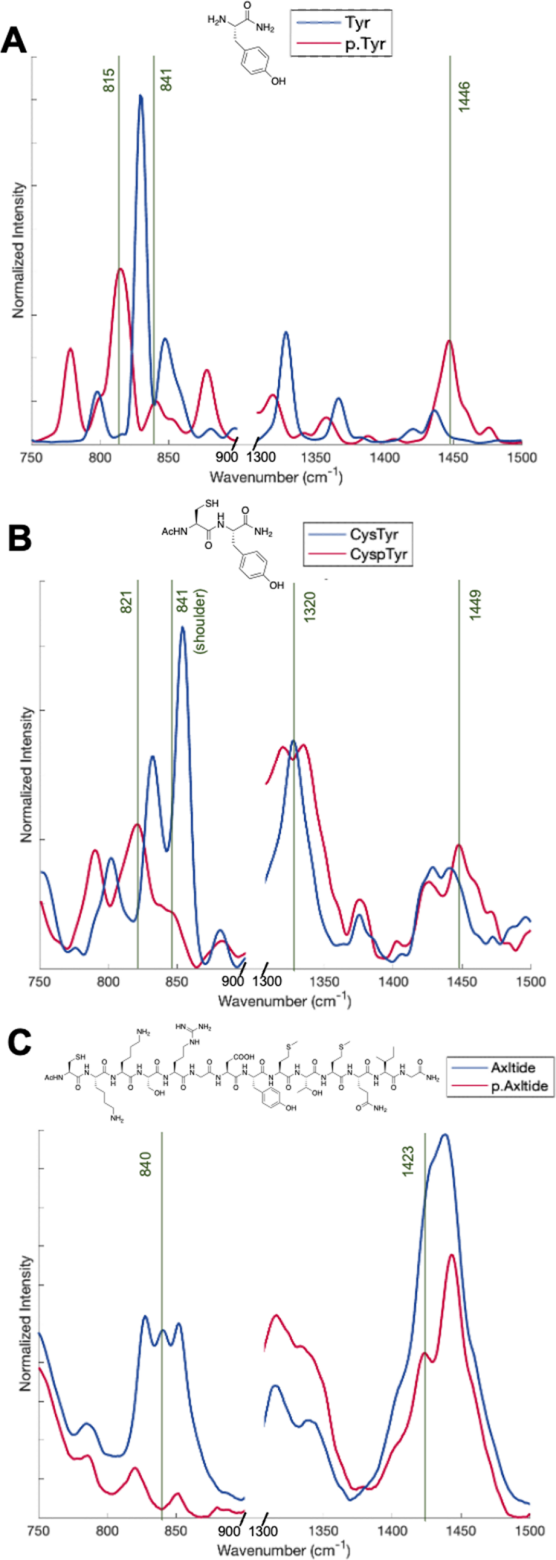
Truncated Raman spectra of solid with relevant peak changes after phosphorylation highlighted with green lines: (A) Tyr (blue) and p.Tyr (red) amino acids; (B) Cys–Tyr (blue) and Cys-p.Tyr (red) dipeptides; and (C) Cys-Axltide (blue) and p.Cys-Axltide (red) peptides.

Prior to assembling the peptide onto the microsensor, we characterized the spontaneous Raman spectra of the Cys-Axltide peptide, l*-*tyrosine, and Cys–Tyr dipeptide, the last of these being a simple model that incorporates both the reactive hydroxyl group and the thiol used for surface attachment. Figure 2A shows the Raman spectra of l-tyrosine with respect to phospho-l-tyrosine (p.Tyr) where the main feature differences were: (1) the collapse of the doublet at 848/829^35,36^ to a singlet at 841 cm^–1^ and (2) the presence of the large peak at 815 cm^–1^, which is likely due to an O–P–O stretch.^37^ Additionally, we observed a large increase and upfield shift of the peak at 1446 cm^–1^ for p.Tyr accounting for CH_2_ vibrations. These findings correlate well with Abramczyk et al.^38^ (full spectra and peak assignment can be found in Figure S1 and Table S1). In the Cys–Tyr dipeptide spectra in Figure 2B, we observe a similar change in peak pattern at 854/832 which, upon phosphorylation, appears to collapse into a shoulder peak of the O–P–O peak at 821 cm^–1^ and peak pattern shift of the CH_2_ peaks at 1320 cm^–1^ (singlet to doublet conversion) and 1449 cm^–1^. In the case of Cys-Axltide (Figure 2C), the 820–850 cm^–1^ region again shows a clear pattern change, where after phosphorylation, there is a loss of a peak and an overall large intensity decrease. At higher wavenumbers, we observe intensity changes in phosphorylation between 1300 and 1375 cm^–1^ and a decrease in intensity at 1450 cm^–1^ reveals a new shoulder peak at 1423 cm^–1^.

### SERS Confirms That the Reporter Is Bound to the μS and Identifies the Main Spectral Raman Features

**Figure 3:**
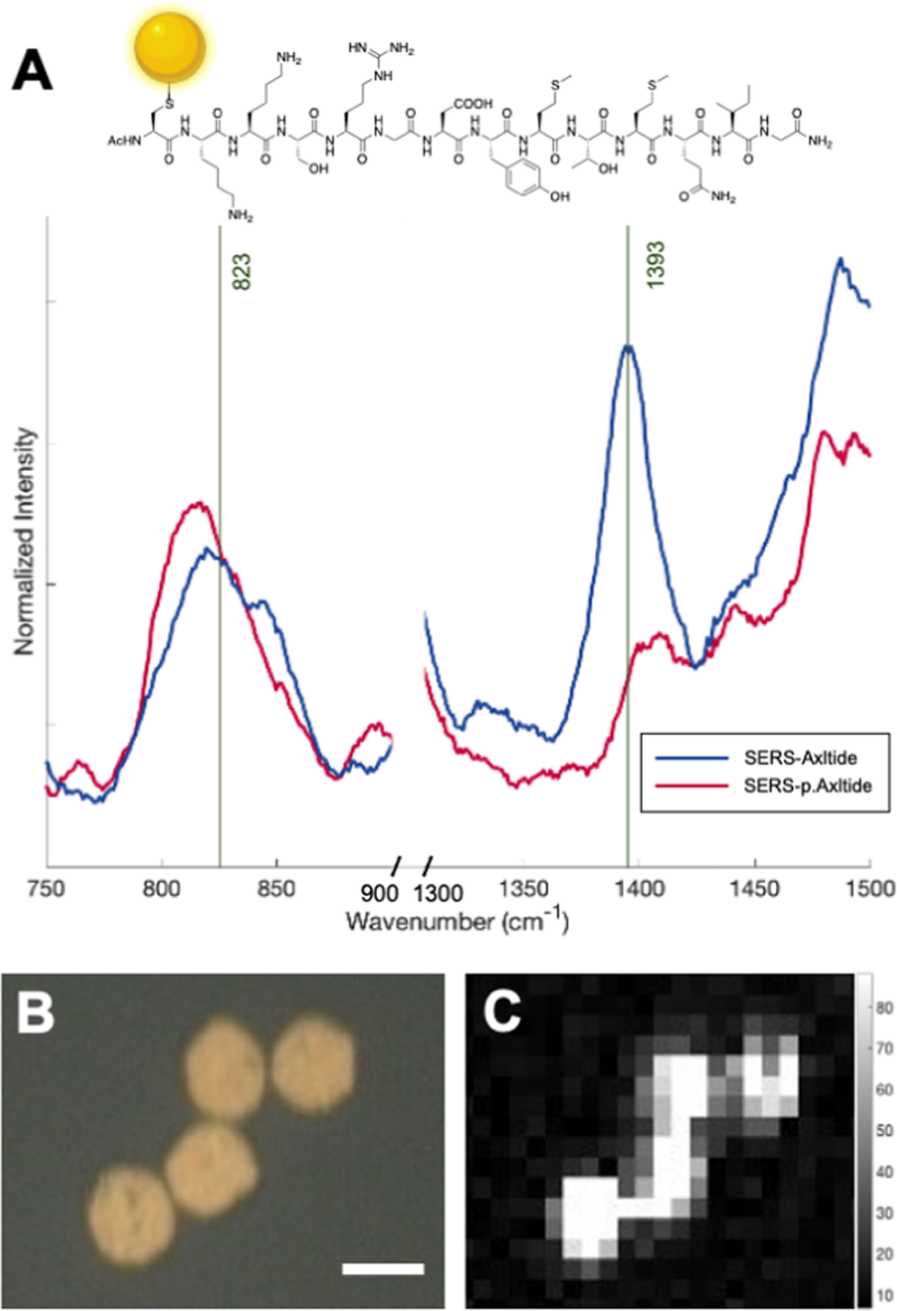
(A) Axltide (blue) and phosphorylated Axltide (red) bound to SERS-μS. (B) White light image of four SERS-Axltide μS. Scale bar: 20 μm. (C) Raman map image showing binding and strong signal localization on the SERS-Axltide microparticles.

SERS spectra are often different from their conventional Raman spectral counterparts due to intensity changes and slight wavenumber shifts from the SERS effect, so assigning SERS spectra is not always simple.^39−41^ The Cys-Axltide and p.Cys-Axltide were incubated with SERS microsensors (μS) and spectra were obtained. While there are features inherent to the μS in the p.Axltide full spectrum (e.g., at 1028 and 1000 cm^–1^, Figure S2), key peaks observed in the regions at 750–900 and 1300–1500 cm^–1^ in Figure 2 are still prominent, indicating good binding of the substrates to the μS. In the Axltide SERS spectra, there is a clear intensity decrease upon phosphorylation in the peak at 1393 cm^–1^, which accounts for a CH_2_ deformation vibration.^42,43^ Again, an increase and shape change is apparent in the peak at 819 cm^–1^, which is likely due to the O–P–O stretch,^37^ as identified in Figure 2A (both of these modes are illustrated in Figure S7). Additionally, a Raman map was taken to demonstrate that the SERS signal is localized on the SERS-μS (Figure 3B). To maximize consistency between μS and their associated spectra, thiol capping of the gold was tested as has previously been proven to improve performance.^44−46^ Increased stability and spectral consistency were observed after incubation with 1 mM mercaptoethanol (MCE) (Figure S4) and, therefore, MCE capping was used in subsequent experiments.

### Using SERS-Axltide to Monitor Phosphorylation in Real Time

An HPLC-MS assay was carried out to confirm the efficacy of AXL enzyme to phosphorylate the cys-Axltide peptide and determine concentrations of enzyme and peptide needed for a relevant reaction time scale. In these experiments, 250 μM Axltide was >75% phosphorylated over a period of 24 h (Figure S5). Using this concentration of enzyme, we carried out an experiment to determine whether the same spectral differences observed in Figure 3A between Axltide and p.Axltide could be seen when Axltide was enzymatically phosphorylated on the surface of the μS (Figure 3A). Again, we found that the peak at 823 cm^–1^ increased upon phosphorylation and the peak at 1393 cm^–1^ decreased. Furthermore, as these peaks exhibit opposite intensity changes, the corresponding ratio of their intensities increases upon phosphorylation, removing any variability resulting from changes in absolute intensity (Figure 3A). This ratio displayed a nearly 60% increase between Cys-Axltide and p.Cys-Axltide, indicating that these were key peaks for monitoring the phosphorylation of Axltide on SERS-μS.

**Figure 4:**
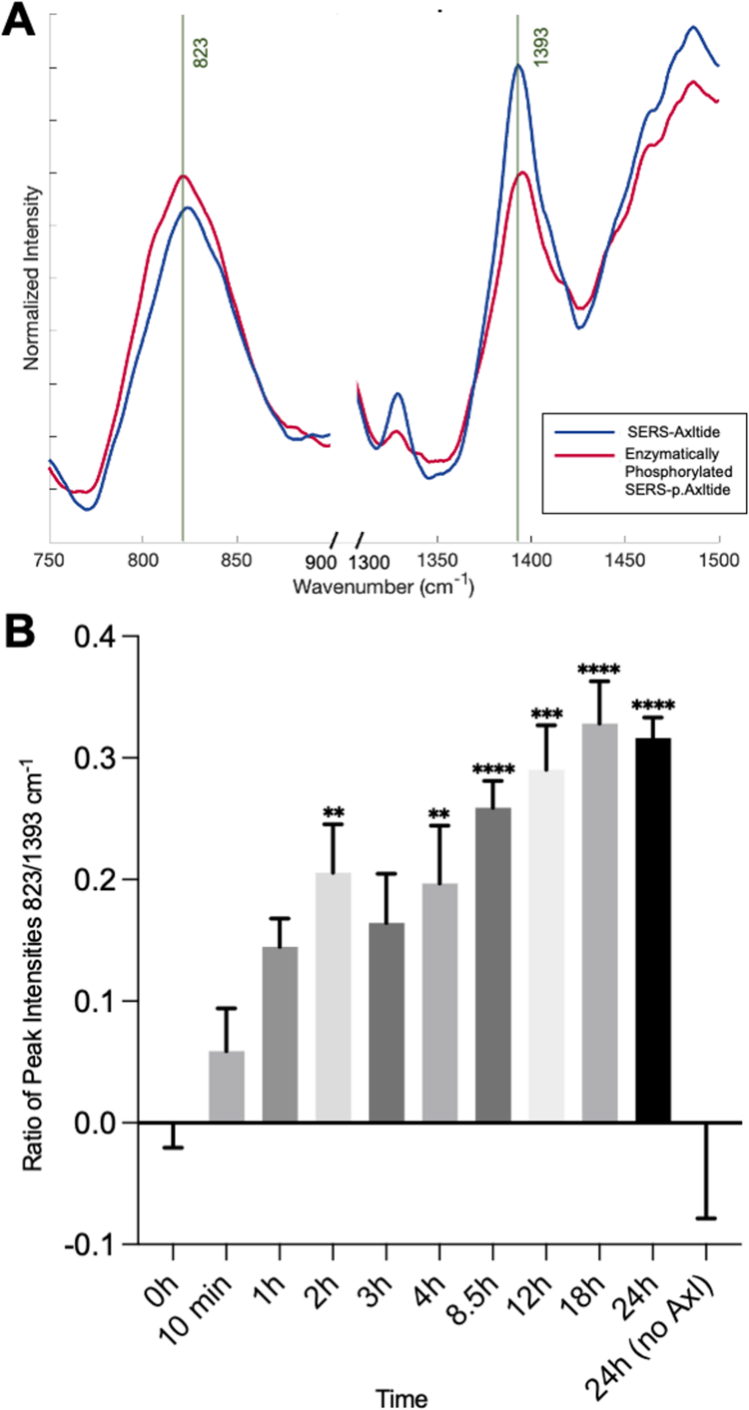
(A) Stacked SERS-Axltide Raman spectra of SERS-Axltide and enzymatically phosphorylated after 24 h in Axl assay conditions with lines (green) to show key peaks. (B) Graph showing intensity ratio of peaks at 823/1393 relative to 0 h demonstrating a significant increase in signal over time. Statistical significance compared to time 0 was determined with one-way ANOVA with Dunnett’s test where **p < 0.01, ***p < 0.001, ****p < 0.0001. *N* = 5.

Finally, we investigated whether the ratio of the peaks at 823 and 1393 cm^–1^ could be used to monitor the phosphorylation of Axltide on SERS-μS in real time. From Figure 4A, the peak at 1393 cm^–1^ clearly decreases over time with phosphorylation and the peak at 823 cm^–1^ remained steadier, although increased as expected (Figure S7). The other peaks in the SERS-Axltide spectra varied little over the 24 h reaction time (Figure S6). When the intensity of each of the 823 and 1393 cm^–1^ peaks at each time point was calculated and the ratio plotted, it was observed that the 823/1393 peak ratio increased significantly over time and evened out around 24 h. By the 2 h point, the peak ratio was significantly different to the starting point where *p* < 0.01. At 24 h without enzyme, the peaks remained the same as at 0 h. Figure 4B clearly shows the time-dependent phosphorylation of Axltide, demonstrating the utility of the SERS-Axltide microparticles in ratiometrically assessing this phosphorylation in real time.

## Conclusions

We have successfully synthesized a version of the Axltide peptide with an *N*-terminal cysteine that can be attached to a gold surface and used in a sensor of phosphorylation. Spectral differences between phosphorylated and nonphosphorylated peptides were determined both as solid and SERS spectra, and we highlighted the peaks at 1393 and 820 cm^–1^ to be the most crucial in the use of our microsensor. We demonstrated that SERS-Axltide can be used to report quantitatively on enzyme activity in an assay that requires no sample preparation and can be read in real time. While we have demonstrated the applicability to making measurements in a well-plate format we believe that the sensor could be adapted to making measurements in a tissue microenvironment. Furthermore, we believe that there is scope to develop other peptide-based sensors of enzyme activity, e.g., we might expect to see pronounced changes on the phosphorylation of other amino acids such as histidine.

## Supporting Information Available

Additional experimental details, full spectra, LC-MS assay, and stability test figures (PDF)
