# Supplementary material for: Label-Free SERS Sensors for Real-Time Monitoring of Tyrosine Phosphorylation
Source: Anal Chem. 2024 Oct 29;96(45):17978–83. doi: 10.1021/acs.analchem.4c02860 (PMC11561882; doi:10.1021/acs.analchem.4c02860)
Supplement: Supplementary file 1 — ac4c02860_si_001.pdf [file ac4c02860_si_001.pdf]

Supporting Information

Label-free SERS sensors for real-time monitoring of tyrosine phosphorylation

Ailsa Geddis[a,b,c], Lorena Mendive-Tapia[b,c], Audreylia Sujantho[a,b], Erica Liu[a,b], Sarah McAughtrie[a†], Richard Goodwin[d], Marc Vendrell[b,c], Colin J. Campbell[a,c*]

[a]EaStCHEM School of Chemistry, The University of Edinburgh, EH9 3FJ, Edinburgh, U.K;

[b] Centre for Inflammation Research, The University of Edinburgh, EH16 4UU Edinburgh, U.K;

[c] IRR Chemistry Hub, Institute for Regeneration and Repair, The University of Edinburgh, EH16 4UU Edinburgh, U.K.

[d] Clinical Pharmacology and Safety Sciences, Biopharmaceuticals R&D, AstraZeneca, Cambridge, CB4 0WG, United Kingdom

*colin.campbell@ed.ac.uk

Table of Contents

1. **Materials and Methods**
    1.1. Reagents and General Methods
    1.2. Peptide Synthesis
    1.3. microBCA assay

2. **Results and Discussion**
    2.1. Description of Optimisation of Experimental Protocols

3. **Figures and Tables**

    Figure S1. Spectral changes of Tyrosine, Cys-Tyr dipeptide and Axltide and their phosphorylated equivalents.

    Figure S2. SERS spectral comparisons of Axltide and p.Axltide.

    Figure S3. Mercaptoethanol and Tentagel® background spectra.

    Figure S4. Stability Test of SERS-Axltide vs SERS-Axltide-MCE.

    Figure S5. HPLC-MS assay.

    Figure S6. Schematic of the optimised Axl Assay.

    Table S1. Detailed table of Raman Peak Assignments.

1.1 Reagents and General Methods. All amino acids and (1-Cyano-2-ethoxy-2-oxoethylidenamino-oxy)dimethylamino-morpholino-carbenium hexafluorophosphate (COMU) were obtained from Iris Biotech GmbH and Bachem AG, TentaGel® R Ram resin (0.18 mmol/g) and TentaGel® M Monosized Amino Microspheres (20 µm) from Rapp Polymere GmbH and ethyl cyano(hydroxyamino)acetate (Oxyma) from CEM Chemicals. Piperidine, piperazine, acetic anhydride, diethyl ether, DL-dithiothreitol (DTT) and N,N-diisopropylcarbodiimide (DIC) and triisopropylsilane (TIS) from Acros Organics, dimethylformamide (DMF), dichloromethane (DCM), water, acetonitrile (ACN) from VWR International and trifluoroacetic acid Peptide Grade (TFA) from Fluorochem Ltd. Gold nanoparticles (150 nm, stabilised suspension in citrate buffer) were obtained from Sigma Aldrich.

1.2 Synthesis of Peptides. Syntheses were carried out under microwave conditions on a 0.05 mmol scale and 5-fold excess of reagents. 0.2 M solutions of all Fmoc-protected amino acids were prepared, along with solutions of DIC (0.25 M), Oxyma (0.5 M) and piperazine (5% (w/v) with 0.1 M Oxyma in DMF) for coupling. 0.278 g TentaGel® R RAM resin (0.18 mmol/g) was used for all syntheses.

[Cys-Tyr] CY. Tyr was coupled for 3 minutes at max 90 ^{0}C and deprotected for 1 minute at 75 ^{0}C. Cys was coupled for 2 minutes at 25 ^{0}C and 8 minutes at 50 ^{0}C. Final deprotection was carried out for 2 minutes at max 90 ^{0}C.

[Cys-p.Tyr] CpY. Double coupling was done for p.Tyr and Cys, each coupling for 2 minutes at 25 ^{0}C and 8 minutes at 50 ^{0}C. P.Tyr was deprotected for 1 minute at 75 ^{0}C and Cys was deprotected for 2 minutes at max 90 ^{0}C.

[Cys-Axltide] CKKSRGDYMTMQIG. The first 7 amino acids (Tyr-Met-Thr-Met-Gln-Ile-Gly-NH_2) were deprotected for 2 minutes and coupled for 3 minutes at max 90 ^{0}C. Amino acids from Asp onwards were deprotected for 1 minute at 75 ^{0}C. Double coupling was carried out for Arg, each for 3 minutes at max 90 ^{0}C. Cys was coupled for 2 minutes at 25 ^{0}C and 8 minutes at 50 ^{0}C. Final deprotection was carried out for 2 minutes at max 90 ^{0}C.

[Cys-p.Axltide] CKKSRGDpYMTMQIG. All amino acids before phosphorylated Tyr (Met-Thr-Met-Gln-Ile-Gly-NH_2) were coupled using the automated synthesizer, under the same conditions as the other peptides, with 2 minutes deprotection and 3 minutes coupling both at maximum 90 ^{0}C. Coupling of p.Tyr and following amino acids were carried out manually with SPPS. Resin was rinsed with DMF (x4), DCM (x3) and DMF (x4). Coupling was then conducted under basic conditions with 1.5 equiv Fmoc-Tyr(PO(OBzl)OH)-OH, 1.5 equiv COMU, 1.5 equiv Oxyma and 3 equiv DIPEA for 1 hour at RT. Kaiser test was performed to ensure complete coupling. Deprotection for all amino acids was done by treatment with 20% piperidine + 1M Oxyma in DMF for 1 x 1 minute, followed by 2 x 5 minutes. Resin was rinsed before the next coupling. For the rest of the amino acids, basic coupling was carried out using amino acid (4 eq), COMU (4 eq), Oxyma (4 eq) and DIPEA (8 eq) for 1 hour at RT. Three couplings were done for Asp as the Kaiser test showed incomplete coupling after the first two. Single coupling was done for Gly, Arg, Ser, Lys and Lys. For the last amino acid, Cys, the first two couplings were done under neutral conditions with amino acid (4 eq), Oxyma (4 eq) and DIC (4 eq) for 1 hour at RT. As the Kaiser test still showed incomplete coupling, the third one was done under basic conditions.

After deprotection of all peptides, they were acetylated using DIPEA (10 eq) and acetic anhydride (10 eq) in DMF for 30 minutes at RT. Resin was rinsed before cleaving with TFA/TIS/H_2O/DTT solution (93:2:5:5 w/v) for 1 hour at room temperature. For Cys-Tyr and Cys-p.Tyr, the cleaving solution was evaporated and the remaining solution was lyophilised. *Cys-Tyr:* LC/MS (m/z): calcd for $C_{14}H_{19}N_3O_4S$ $[M+H]^+$: 326.11, found: 326.02. *Cys-p.Tyr:* LC/MS (m/z): calcd for $C_{14}H_{20}N_3O_7PS$ $[M+H]^+$: 406.08, found: 405.94.

For Cys-Axltide and Cys-p.Axltide, the cleaving solution was then evaporated and ice cold diethyl ether added to form precipitate. Precipitate were dissolved in H_2O/ACN (1:1) solution and lyophilised. Crude peptides were then purified using preparative HPLC. Cys-*Axltide:* HRMS ESI (m/z): calcd for $C_{68}H_{115}N_{21}O_{21}S_3$ $[M+H]^+$: 1657.77, found: 1658.560. Cys-*p.Axltide:* HRMS ESI (m/z): calcd for $C_{68}H_{116}N_{21}O_{24}PS_3$ $[M+H]^+$: 1738.75, found: 1738.566.

1.3 MicroBCA Assay. Protocol taken from ThermoScientific Manual 23235. Albumin (BSA) standard (2.0 mg/mL) was diluted to 200, 40, 20, 10, 5, 2.5, 1 and 0.5 μg/mL and Micro BCA working reagent (WR) was made according to protocol (Reagents MA:MB:MC, 25:24:1). A range of unknown samples were made up: 5 mM solution of Axltide, diluted 2 μL in 1 mL, supernatant left over from AuTG bind, diluted 2 μL in 1 mL, and SERS-Axltide beads in 400 μL water, diluted 5 μL in 145 μL. In a 96 well plate, 150 μL of standard or unknown sample and 150 μL WR were added and shaken thoroughly for 30 seconds. Plate covered and incubated at 37 °C for 2 hours, cooled to rt and absorbance at 562 measured to obtain a standard curve of the micro BCA, plotted in GraphPad Prism. Unknown samples compared to the standard curve to find the concentration of peptide bound to TG Beads.

2. Results and Discussion

2.1 Optimisation of Experimental Protocols. Having demonstrated that the peptide binds to the SERS-MS we found the amount of peptide bound using a microBCA assay to be 178 nmol per mg of SERS-MS. To enhance the performance of the SERS detection, so we incubated our uncapped SERS-Axltide microparticles with 1 mM mercaptoethanol (MCE) for 2 hours and thoroughly washed. Stability testing showed high consistency of the spectra over 7 days and most importantly the same spectral peaks at ~820 cm^{-1} and ~1400 cm^{-1} were still visible and different on phosphorylation (Figure S4). Finally, a rigorous method for obtaining and then processing the SERS spectra with MATLAB was developed to ensure maximum data inclusion such that of the 5 MS per technical replicate, 60-120 spectra were taken resulting in 3-500 spectra per replicate and after removing saturated spectra and spectra with no significant signal, more than 60% of the data per MS was included (code available on request).

3. Figures

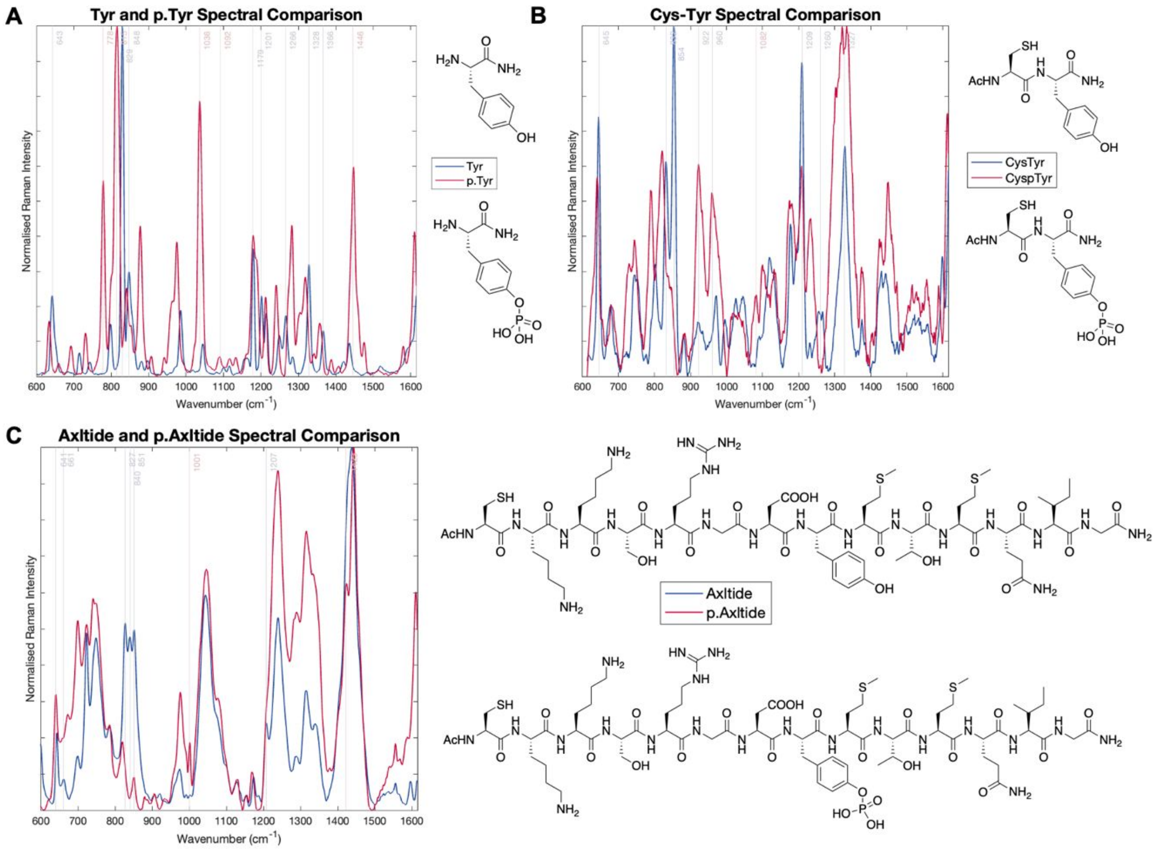

Figure S1. Spectral Comparisons (with structures on RHS) of **A** tyrosine (blue) and p-tyrosine (red) amino acids; **B** Cys-Tyr (blue) and Cys-p-Tyr (red) solid dipeptides; and **C** Axltide (blue) and phosphorylated Axltide (red) solid peptides.

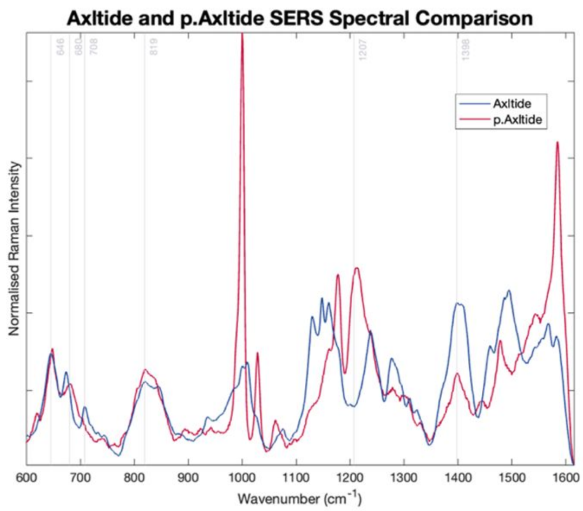

Figure S2. Spectral comparisons of Axltide (blue) and phosphorylated Axltide (red) bound to SERS-MS.

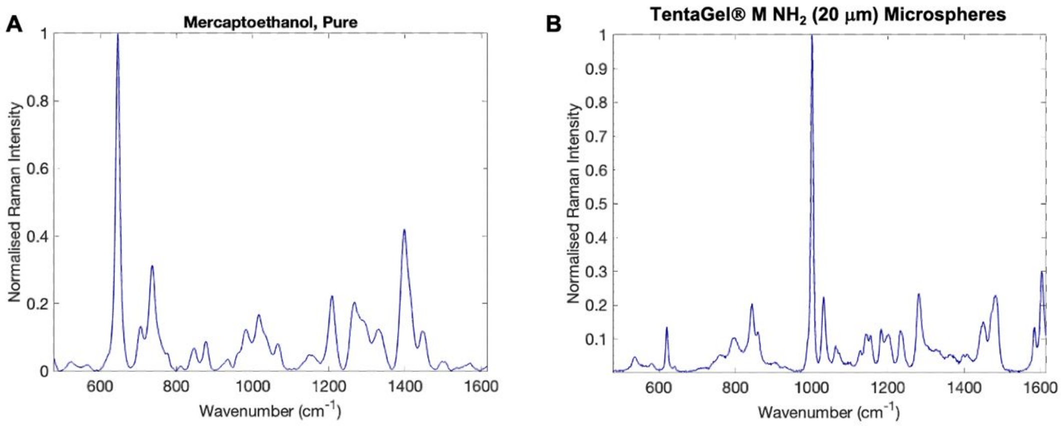

Figure S3. Relevant background spectra, **A** Mercaptoethanol spectrum; **B** Tentagel Spectrum®, 20 μm Amino monosized microspheres.

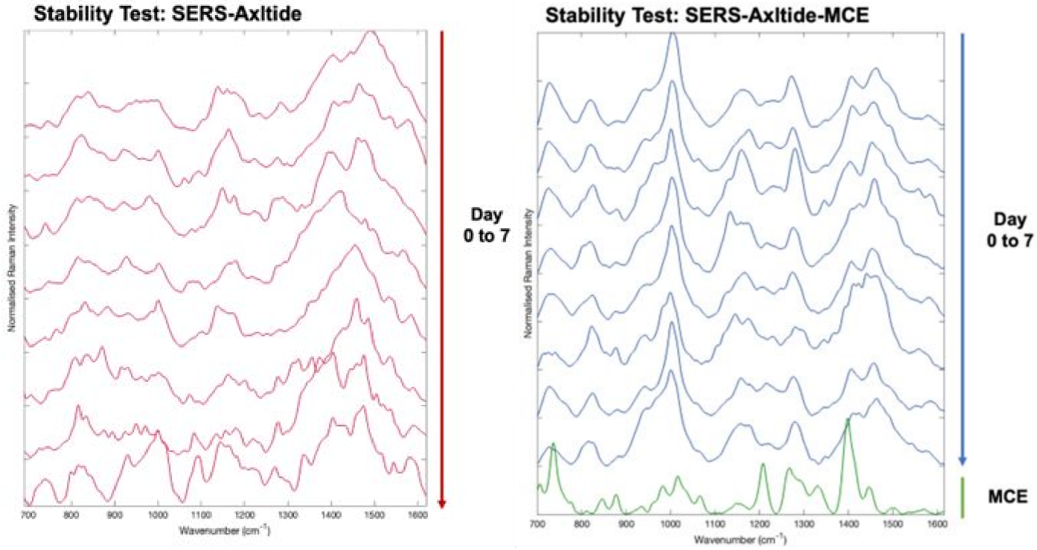

Figure S4. Stability test of SERS-Axltide in PBS buffer over 7 days vs SERS-Axltide-MCE in PBS buffer over 7 days with Mercaptoethanol spectrum to demonstrate peak are particular to the Axltide not the MCE. Spectra offset for clarity.

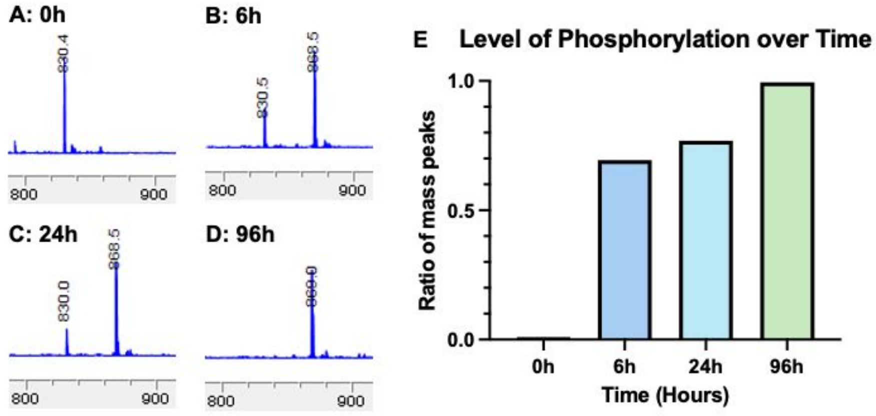

Figure S5. HPLC-MS Assay **A-D** MSD1 TIC traces at 0h-96h respectively. Axltide expected: 1658; observed: m/z 830 ($M^+/2$). p.Axltide expected: 1737; observed m/z 869 ($M^+/2$). **E** Ratio of mass peaks at 869/830 showing phosphorylation height increasing with time, n = 1.

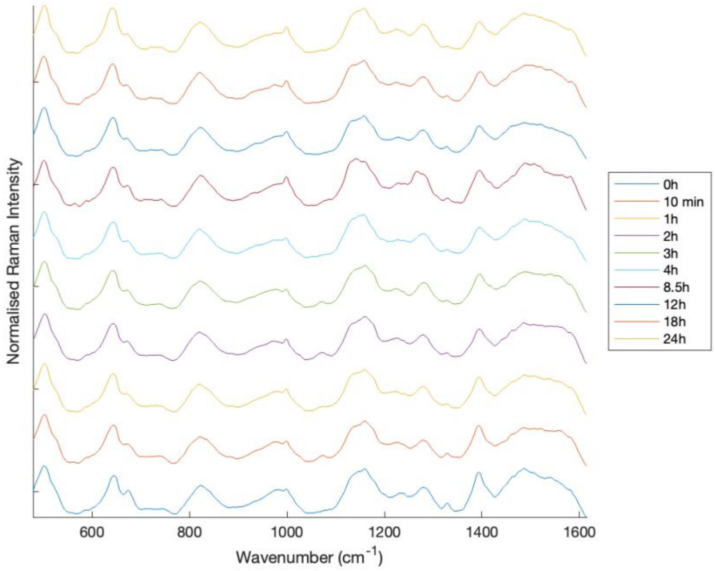

Figure S6. Average SERS-Axltide spectra from each time point of the Axl assay over full 24 hour time period.

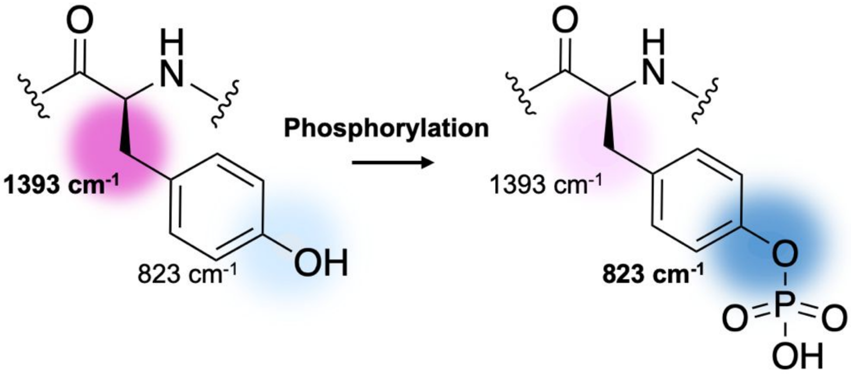

Figure S7. Schematic highlighting the key peak changes after phosphorylation of Tyrosine which correspond to the key changes observed in the SERS spectra and in the final assay (similar peaks observed in the spontaneous spectra albeit with a slight left peak shift). The peak at 1393 cm^{-1} corresponds to a CH_2 deformation of the CH_2 para to the O-H to O-PO_3H transformation with intensity decrease after phosphorylation and the peak at 823 cm^{-1} corresponds to the O-H to O-PO_3H transformation.

Table S1. Raman Peak Assignments

Peak assignments of key analytes. Peaks are grouped in similar regions of wavenumbers and assigned specifically where possible. Grey regions of the table are the phosphorylated compounds. Peaks in blue are assigned as residual tentagel peaks so may not follow patterns observed in other assignments.

**Vibrational Mode**	**C=C bending/ stretching**				**CH_2 Deformation of the Ar adjacent CH_2**	**CH_2/CH_3 twisting / wagging**			**Amide III / C-H in plane bending**			**OH**	**In-plane bending**		**OPO anti sym Str 1090**	**OPO sym str 1070**			**OH hydrogen bonding state**	**OPO vib**			**C-C twisting**
What happens on phosphorylation?	Shift →				Intensity ↓					Shift →									Fermi Doublet Collapse / Intensity change				Shift α Or β
Tyr (Fig 2A)	**1615**			1420		**1366**	**1328**			**1266**	1214	**1201**	**1179**			1044	985		**848/829**		798		**642**
pTyr (Fig 2A)	**1610**	**1580**	**1446**			**1357**	**1318**	1304	1282	**1241**	1212		**1179**		**1089**	**1036**	975	877	**841**	**815**	778	730	**634**
Cys-Tyr (Fig 2B)	**1618**	**1598**	**1441**	**1430**		**1375**	**1327**			**1260 d**	**1209**		**1177**	**1118**	**1045**	**1026**	**972**	**882**	**854/ 832**	**802**		**743**	**645**
Cys-pTyr (Fig 2B)	**1612**	**1588**	**1448**	**1424**	**1404 / 1392**	**1374**	**1333**	**1320**		**1233**	**1209**		**1175**	**1132 1100**	**1082**	**1057**	**958**	**884**	**821**		**789**	**743, 730**	**641**
Axltide (Fig 2C)	1617	**1596**	1555	1438		1340	1315		1286	1240	**1207**		1174	1153	1129	1044	974		**851/840/ 827**		785	749, 723, 700	661/644
pAxltide (Fig 2C)	1611	1555	1443	**1423**			1316		1289	1239			1168	1155 1131		1046	**1001** 976		852/820		786	741, 723, 700	672/641
Axltide (SERS) (Fig 3A)	1583	1568	1494	1459	1398				1277	1238			1161	1149 1130	1075		1009	936	843/ 819			708	674/646
pAxltide (SERS) (Fig 3A)	1585	**1543**	1478	1445	**1398**			**1295**	1278		1211		1177		1062	1028	1000		**819**				**680/649**
Tentagel	**1604**	**1585**	**1482**	**1449**					1282	1232	1201	1182	1154	1142	1063	**1032**	**1001**		**844**		797		620
